# Natural Killer Cell Recruitment to the Lung During Influenza A Virus Infection Is Dependent on CXCR3, CCR5, and Virus Exposure Dose

**DOI:** 10.3389/fimmu.2018.00781

**Published:** 2018-04-17

**Authors:** Lindsey E. Carlin, Emily A. Hemann, Zeb R. Zacharias, Jonathan W. Heusel, Kevin L. Legge

**Affiliations:** ^1^Interdisciplinary Graduate Program in Immunology, Department of Pathology, University of Iowa Carver College of Medicine, Iowa City, IA, United States; ^2^Department of Pathology and Immunology, Washington University School of Medicine, Saint Louis, MO, United States; ^3^Department of Microbiology and Immunology, University of Iowa Carver College of Medicine, Iowa City, IA, United States

**Keywords:** natural killer cells, influenza virus, cell trafficking, lung, CXCR3, CCR5

## Abstract

Natural killer (NK) cells are vital components of the antiviral immune response, but their contributions in defense against influenza A virus (IAV) are not well understood. To better understand NK cell responses during IAV infections, we examined the magnitude, kinetics, and contribution of NK cells to immunity and protection during high- and low-dose IAV infections. Herein, we demonstrate an increased accumulation of NK cells in the lung in high-dose vs. low-dose infections. In part, this increase is due to the local proliferation of pulmonary NK cells. However, the majority of NK cell accumulation within the lungs and airways during an IAV infection is due to recruitment that is partially dependent upon CXCR3 and CCR5, respectively. Therefore, altogether, our results demonstrate that NK cells are actively recruited to the lungs and airways during IAV infection and that the magnitude of the recruitment may relate to the inflammatory environment found within the tissues during high- and low-dose IAV infections.

## Introduction

Natural killer (NK) cells, innate lymphocytes with cytolytic activity against infected and transformed cells, have known roles in protection from DNA virus infections, including members of the herpesviridae, poxviridae, and papillomaviridae families (1–3). More recently, NK cells have been shown to participate in the immune response against the orthomyxovirus influenza A virus (IAV), whose segmented genome is composed of negative sense RNA. However, it is currently less clear as to what contribution NK cells play [i.e., protective (4–11) vs. immunopathologic (12, 13)] in immunity during IAV infections. While differences in animal models, virus strains, route of infection, and dose of virus may explain the discrepancies between protective and immunopathologic effects, overall, these studies all suggest that NK cells are a component of normal immunity to IAV.

Natural killer cell-mediated protection from infections requires effective NK cell recruitment to the sites of lymphocyte activation and infection. NK cell recruitment to the liver is critical for the control of both *Toxoplasma gondii* and murine cytomegalovirus, while recruitment to the draining lymph nodes (DLNs) in ectromelia virus infection is important for priming an effective CD8 T cell response (14–16). These data suggest that proper NK cell trafficking is important for both the initial control of infections at the replication site and the subsequent priming of an adaptive immune response against the pathogen. Currently, the mechanisms and molecular networks controlling NK cell trafficking and accumulation in the lungs and draining lymphatics during IAV infection remain unclear. In general, NK cells express many chemokine receptors, including CCR2, CCR5, and CXCR3 that have been linked to NK cell migration (17). In the lung tissue, CCR2 has been shown to be important for NK cell recruitment and subsequent protection from invasive *Aspergillus fumigatus* infections (18). While CCR2 has been shown to influence NK cell accumulation in the IAV-infected airway, its absence had no effect on NK cell recruitment to the IAV-infected lung parenchyma (19). This suggests that the mechanism of NK cell recruitment may differ between pulmonary infections. CXCR3 is known to be important in NK cell recruitment to the lung in homeostasis, as CXCR3^−/−^ mice have significantly fewer NK cells in the lungs than WT mice (20). While NK cell expression of CXCR3 can result in an increased NK cell accumulation in the lungs during pulmonary inflammation (20, 21), the importance of CXCR3 in recruiting NK cells to the lung during IAV infection has not yet been determined. In addition to recruitment to individual organs, chemokine receptors can localize cells within an organ. For example, while CXCR3 expression is important in CD8 T cell recruitment to the lung, CCR5 expression on CD8 T cells is required for the localization of memory CD8 T cells to the IAV-infected epithelium (22, 23). While it has been shown that CCR5^−/−^ NK cells are better able to proliferate in the IAV-infected lung compared to those in WT (24), the role of CCR5 in NK cell recruitment to and localization within the IAV-infected lung has not been directly examined.

As the dose of virus may affect how NK cells contribute to IAV immunity, we herein examined if IAV infection dose alters NK cell recruitment to lungs, lung DLNs, and spleen. Given the importance of CXCR3 in NK cell homeostasis, and the role of CXCR3 and CCR5 in recruiting and localizing CD8 T cells to the lung during IAV infection, we specifically determined if CXCR3 and CCR5 are required for NK cell recruitment during both high- and low-dose IAV infections. Our results demonstrate that while NK cells accumulate in the lung and DLN during both high- and low-dose IAV infections, a greater NK cell accumulation occurs in the lungs during high-dose infections and in the DLN during low-dose infections. CXCR3 expression on NK cells increased NK cell recruitment to the lungs, and the increased NK cell recruitment in high-dose IAV infections correlated with a higher expression of CXCR3 ligands in the lungs. CCR5 ligands were also upregulated in the lung and correlated with an increased recruitment of WT NK cells to the lung tissue and airways compared to CCR5^−/−^ NK cells. Overall, our data suggest that in addition to infection-dependent mechanisms of NK cell recruitment to the lung, the severity of infection may also influence the magnitude of NK cell recruitment, thus influencing disease outcome.

## Materials and Methods

### Mice

Six- to eight-week-old BALB/c and C57Bl/6 mice were purchased from the National Cancer Institute (Frederick, MD). BALB.B6-CT6 (i.e., CT6) mice were obtained from Dr. Anthony Scalzo (Nedlands, Australia). CXCR3^−/−^ (B6.129P2-*Cxcr3^tm1Djen^*/J), CCR5^−/-^ (B6.129P2-*Ccr5^tm1Kuz^*/J), and C57Bl/6 CD45.1 (B6.SJL-*Ptprc^a^Pepc^b^*/BoyJ) were obtained from The Jackson Laboratory (Bar Harbor, ME, USA) and bred in house. The C57Bl/6 CD45.1 colony was started prior to 2009 (25). C57BL/6 and C57Bl/6 CD45.1 mice were bred together to generate the CD45.1^+^/CD45.2^+^ mice used in adoptive transfer experiments. All animals were housed, bred, and maintained in the animal care facilities at the University of Iowa. All procedures were approved by the University of Iowa Animal Care and Use Committee.

### Influenza Virus Infections

Mouse-adapted IAV A/PR/8/34 was grown in the allantoic fluid of embryonated chicken eggs as previously described (26). Mice were anesthetized with isofluorane and then infected i.n. with 50 µL of virus diluted in Iscove’s media as indicated. All experiments with IAV were undertaken using BLS2/aBLS2 containment.

### Influenza Virus Titers

Lungs were removed from infected mice at 2, 4, 6, and 8 days post infection (p.i.) and homogenized. Viral titers were determined as previously described by end point dilution assay and expressed as TCIU. Briefly, 10-fold serial dilutions of lung homogenates from IAV-infected mice were mixed with 2.5 × 10^4^ Madin–Darby canine kidney cells incubated at 37°C. After 24 h, culture supernatants were removed and fresh media were added to each well. After 4 days of incubation, culture supernatants were mixed with 0.5% chicken RBC and the agglutination pattern was determined, and the TCIU values were calculated using the Reed–Muench accumulative method.

### NK Cell Depletion

CT6 mice were administered 300 µg of anti-NK1.1 (PK136) monoclonal antibody (mAb) intraperitoneally (i.p.) 2 days before and on the day of IAV infection to deplete NK cells. NK depletion was verified by flow cytometry using mAbs against NKp46 (clone 29A1.4) and CD3ε (clone 17A2).

### NK Cell Purification

Spleens were processed with frosted glass slides to create a single-cell suspension. Lymphocytes were separated from red blood cells using a Ficoll gradient before NK cells were purified using the EasySep Mouse NK Cell Enrichment kit (STEMCELL Technologies, Vancouver, Canada) as per manufacturer’s instructions. For the competitive trafficking assay WT (CD45.1^+^/CD45.2^+^) and CXCR3^−/−^ or CCR5^−/−^ (CD45.2^+^) NK cells were mixed 1:1 before transferring 100 µL i.v. to recipient mice (CD45.1^+^) (1–5 × 10^5^ each NK cell population). Mixed donor cells were analyzed by flow cytometry prior to injection to determine the actual ratio for that individual experiment. Endogenous, transferred WT, and transferred CXCR3^−/−^ or CCR5^−/−^ NK cells were identified by CD45.1 and CD45.2 expression by flow cytometry. For calculations of the ratio of donor NK cell trafficking, the values observed for each of the donor NK cell populations were normalized to the actual input ratio that was observed for the mixed donor cells for that experiment.

### Flow Cytometry

Lungs were digested in DNAse and Collagenase IV for 10 min at 37°C. Organs were processed through a 60-µm screen, and single-cell suspensions were blocked in 2% rat serum. The following antibodies were used for these studies: rat anti-mouse CD3ε (17A2), hamster anti-mouse CD27 (LG.3A10), rat anti-mouse CD11b (M1/70), rat anti-mouse CD49b (DX5), and mouse anti-mouse CD45.2 (104) purchased from BD Biosciences (San Jose, CA, USA); mouse anti-mouse NK1.1 (PK136) purchased from eBioscience (San Diego, CA, USA); and mouse anti-mouse CD45.1 (A20) purchased from Biolegend (San Diego, CA, USA). For surface staining, 10^6^ cells were incubated with antibody for 25 min at 4°C and then fixed in FACS lysis buffer (BD Biosciences, San Jose, CA, USA). All flow cytometry data were collected with a BD Canto II and data analyzed with Flow Jo software (Tree Star, Inc., Ashland, OR, USA).

### NK Cell Proliferation Assay

For proliferation assays, A/PR/8/34-infected BALB/c mice were administered 8 mM carboxyfluorescein succinimidyl ester (CFSE) (50 µL) and 2 h later were given 80 µg bromodeoxyuridine (BrdU) i.n. as previously described (27). Four hours after the BrdU administration, lungs were harvested and single-cell suspensions were prepared for flow cytometric analysis with anti-CD49b (DX5) and anti-CD3ε mAbs as described above. Following cell fixation, BrdU incorporation was determined with the BrdU Flow Kit (BD Biosciences San Jose, CA, USA) according to manufacturer’s instructions. NK cells proliferating within the lung were identified as DX5^+^CD3^−^CFSE^+^BrdU^+^ cells.

### Drug Treatments

For the NK cell trafficking assay, A/PR/8/34-infected BALB/c mice were administered 500 ng pertussis toxin (PT) (Sigma-Aldrich, St. Louis, MO, USA) in 200 µL phosphate buffered saline (PBS) i.p. on days 2, 3, 4, and 5 post IAV infection. For the competitive NK cell trafficking experiment, purified NK cells from C57Bl/6 mice (CD45.2^+^) were incubated with 200 ng/mL PT in RPMI supplemented with 2% FBS for 20 min at 37°C while NK cells from C57Bl/6 mice (CD45.1^+^/CD45.2^+^) were incubated in media + 2% FBS as previously described (28). Cells were washed three times in PBS and then mixed at a 1:1 ratio before transfer. Mixed donor cells were analyzed by flow cytometry to determine the actual input ratio for that individual experiment. For calculations of the ratio of donor NK cell trafficking, the values observed for each of the donor NK cell populations were normalized to the actual input ratio that was observed for the mixed donor cells for that experiment.

### ELISA

Lungs from A/PR/8/34-infected BALB/c and C57Bl/6 mice were harvested at indicated days and were homogenized in 3 mL Iscove’s media, aliquoted, and stored at −80°C. DuoSet ELISA kits (R&D Systems, Minneapolis, MN, USA) were used to determine CCL5, CXCL9, CXCL10, and CXCL11 (BALB/c only) per manufacturer’s instructions.

## Results

### NK Cells Accumulate in the Lung and Lung DLNs During IAV Infection

**Figure 1:**
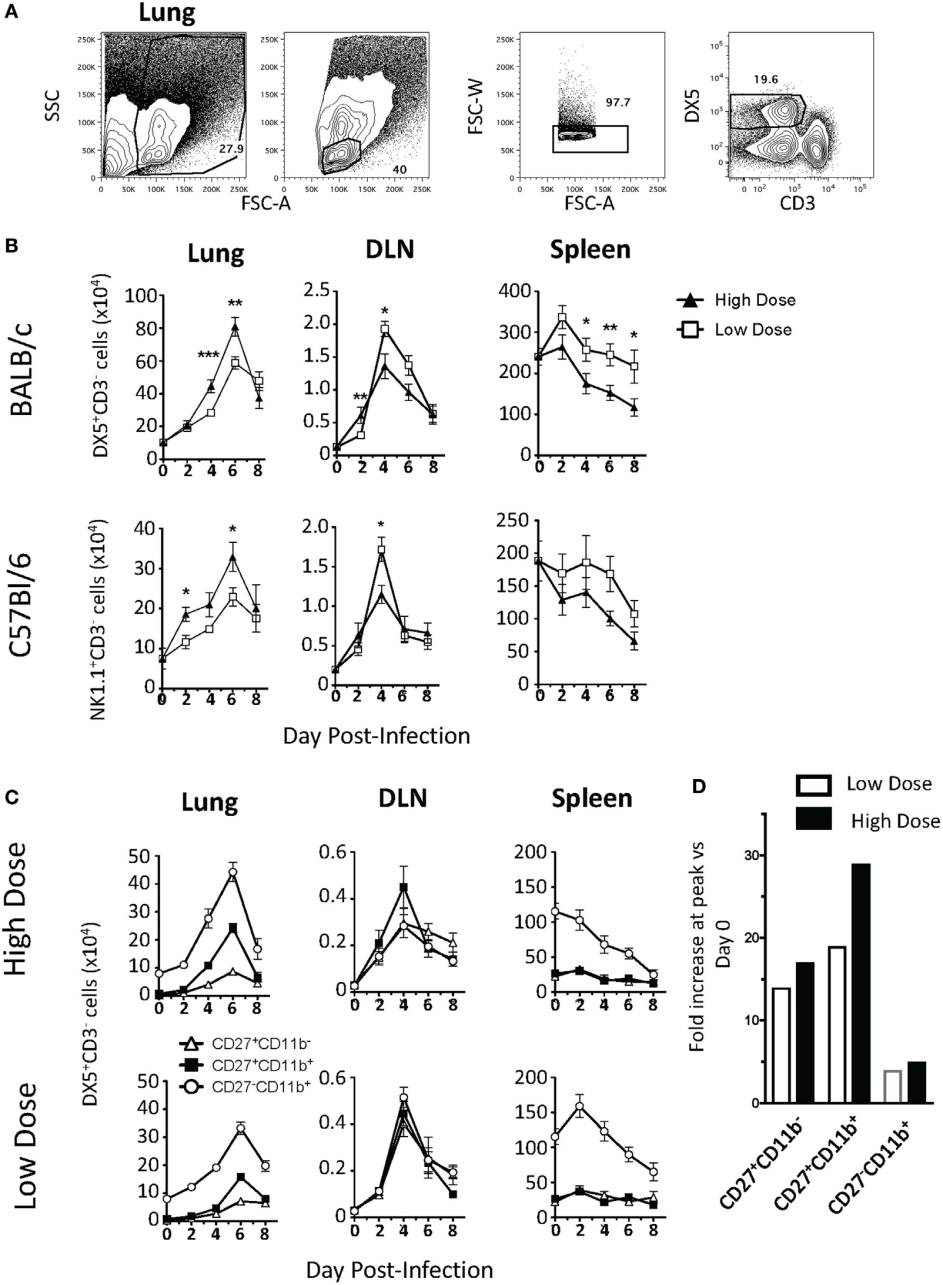
Kinetics of natural killer (NK) cell accumulation during high- and low-dose influenza A virus (IAV) infections. **(A)** Representative gating strategy for DX5^+^CD3^−^ NK cells in the lungs. **(B)** BALB/c (top) or C57Bl/6 (bottom) mice were infected with a high (1 LD_50_) or low (0.1 LD_50_) dose of A/PR/8/34. NK cells were enumerated in the lung, lung-draining lymph node (DLN), or spleen at the indicated time points by flow cytometry. Shown are DX5^+^ or NK1.1^+^CD3ε^−^ cells. The number of NK cells in the DLN is expressed as NK cells per node. **(C)** NK cells (DX5^+^CD3ε^−^ gated) within the lung, DLN, and spleen in BALB/c mice were further characterized by CD27 and CD11b expression to determine the distribution of functional subsets during both high- (top) and low (bottom)-dose IAV infections. Shown are *p*-values for high- vs. low-dose IAV infections. *p < 0.05, **p < 0.005, ***p < 0.001 (two-tailed unpaired Student’s *t*-test). The number of NK cells within the lung of high- and low-dose IAV-infected mice was found to be significantly greater than the number of NK cells in naïve lungs at days 2, 4, and 6 p.i. (*p* < 0.05). The decrease in NK cell numbers in the spleen was found to be significantly greater in high-dose-infected mice by two-way ANOVA (*p* < 0.001). Data are pooled from at least two separate experiments, with five mice per time point, per experiment. **(D)** The fold increase in CD27^+^CD11b^−^, CD27^+^CD11b^+^, and CD27^−^CD11b^+^ NK cells within the lungs was calculated by dividing the cell number found with the lungs at the peak of the response by the cell number found within the lungs at day 0.

Prior studies examining the contribution of NK cells to IAV immunity have shown disparate results; studies using higher infection doses found that NK cells contribute to lethal immunopathology, while studies using lower doses of IAV showed a protective role for NK cells (4–9, 12, 13). Therefore, to determine if the kinetics and location of NK cell trafficking during IAV infection are altered by the severity and initial dose of IAV, BALB/c mice were infected with either a high- (1 LD_50_) or a low (0.1 LD_50_)-IAV dose, and NK cells were subsequently quantified in the lung, lung DLN, and spleen by flow cytometry (see Figure 1A for representative gating strategy). Following infection, the number of NK cells found in the lung was significantly increased at days 2, 4, 6, and 8 p.i. in both the high- and low-dose infections when compared to naïve mice (day 0) (*p* < 0.05, Student’s unpaired *t*-test), with maximal accumulation at day 6 p.i. Moreover, NK cell numbers in the lung were significantly greater in high- vs. low-dose IAV-infected lungs on days 4 and 6 p.i. (Figure 1B, left panel). Within the DLN, the number of NK cells present was significantly higher for IAV-infected mice than in naïve mice at days 2, 4, and 6 p.i. NK cell accumulation peaked at day 4 p.i. for both doses of IAV (Figure 1B, middle panel). However, while NK cell accumulation in the lungs was greater in high-dose IAV-infected mice, accumulation in the DLN was instead significantly higher for the low-dose IAV infection. In the spleen, NK cell numbers initially increased at day 2 p.i. before returning to homeostatic levels in low-dose IAV-infected mice and were significantly decreased relative to naïve mice in high-dose IAV-infected mice (Figure 1B, right panel). In summary, similar kinetics of NK cell trafficking were observed between high- and low-dose IAV infections; however, the overall magnitude of NK cell accumulation differed with a greater NK cell accumulation in the lungs during high-dose IAV and in the DLN during low-dose IAV infection. Importantly, C57Bl/6 mice infected with high and low doses of IAV showed similar kinetics and organ preference in NK cell trafficking as BALB/c mice (Figure 1B), suggesting that the pattern of NK cell trafficking during IAV infection was not mouse strain-specific.

Natural killer cells can be subdivided into subsets by CD27 and CD11b expression (CD27^+^CD11b^−^, CD27^+^CD11b^+^, and CD27^−^CD11b^+^) to correspond to different stages of maturation (29). These NK cell subsets differ in effector molecule expression as well as chemokine receptor expression (17, 29, 30). Therefore, differential recruitment of the various NK cell subsets could correlate with their different roles in immunity (31) or in protection vs. immunopathology during IAV infection. When we quantified the recruitment of NK cell subsets within the lung, DLN, and spleen of high- and low-dose IAV-infected mice, we observed increases in all three NK subsets in the lung and DLN. In the lung, mature CD27^−^CD11b^+^ NK cells accumulated in the greatest number in both the high- and low-dose infections (Figure 1C); however, the intermediate CD27^+^CD11b^+^ subset showed the greatest relative increase in magnitude (Figure 1D). The immature CD27^+^CD11b^−^ NK cells also increased in number within the infected lung, but showed no significant difference between high- and low-dose infections (*p* > 0.05) (Figure 1C, left panels), suggesting that the CD27^+^CD11b^−^ NK cell subset did not contribute to the overall difference in NK cell numbers observed in the lungs between high- and low-dose IAV infections. Similar to studies utilizing a single dose of IAV (32), the accumulation of all three NK subsets was also observed in the DLN of both high- and low-dose IAV-infected mice. NK cell subsets were evenly distributed within the DLN, and all NK cell subsets had a maximal accumulation at day 4 p.i. (Figure 1C, middle panels)—a kinetics which is in excellent agreement with those recently reported (31). Unlike what was observed in the lung and DLN, where all NK cell subset numbers increased within the tissues, only the CD27^−^CD11b^+^ NK cell subset had altered kinetics in the spleen during the course of both high- and low-dose IAV infection. The number of CD27^−^CD11b^+^ NK cells decreased in both infection doses (Figure 1C, right panels). Notably, the CD27^−^CD11b^+^ subset had the greatest increase in the lung, while it had the greatest decrease within the spleen, suggesting that NK cell accumulation within the lung during IAV infection may be due, in part, to recruitment of this NK cell subset from the spleen.

As expected, based on our prior studies (26), despite the differing accumulation level of NK cells during high- and low-dose infections, we observed no differences in IAV virus titers in the lungs on day 2, 4, 6, or 8 p.i. (Data Sheet S1 in Supplementary Material). Further, when we depleted NK cells from BALB.CT6 mice (BALB/c mice that express NK1.1), we did not observe changes in overall morbidity and mortality (Data Sheet S1 in Supplementary Material). This suggests that in the absence of NK cells, or when infection conditions lead to differing magnitudes of NK cell accumulation within lungs (Figure 1), other immune cell populations may compensate to control virus loads and dictate the outcome of the infection.

### NK Cell Accumulation in the Lung During IAV Infection Is Not due to Apoptosis or Proliferation

**Figure 2:**
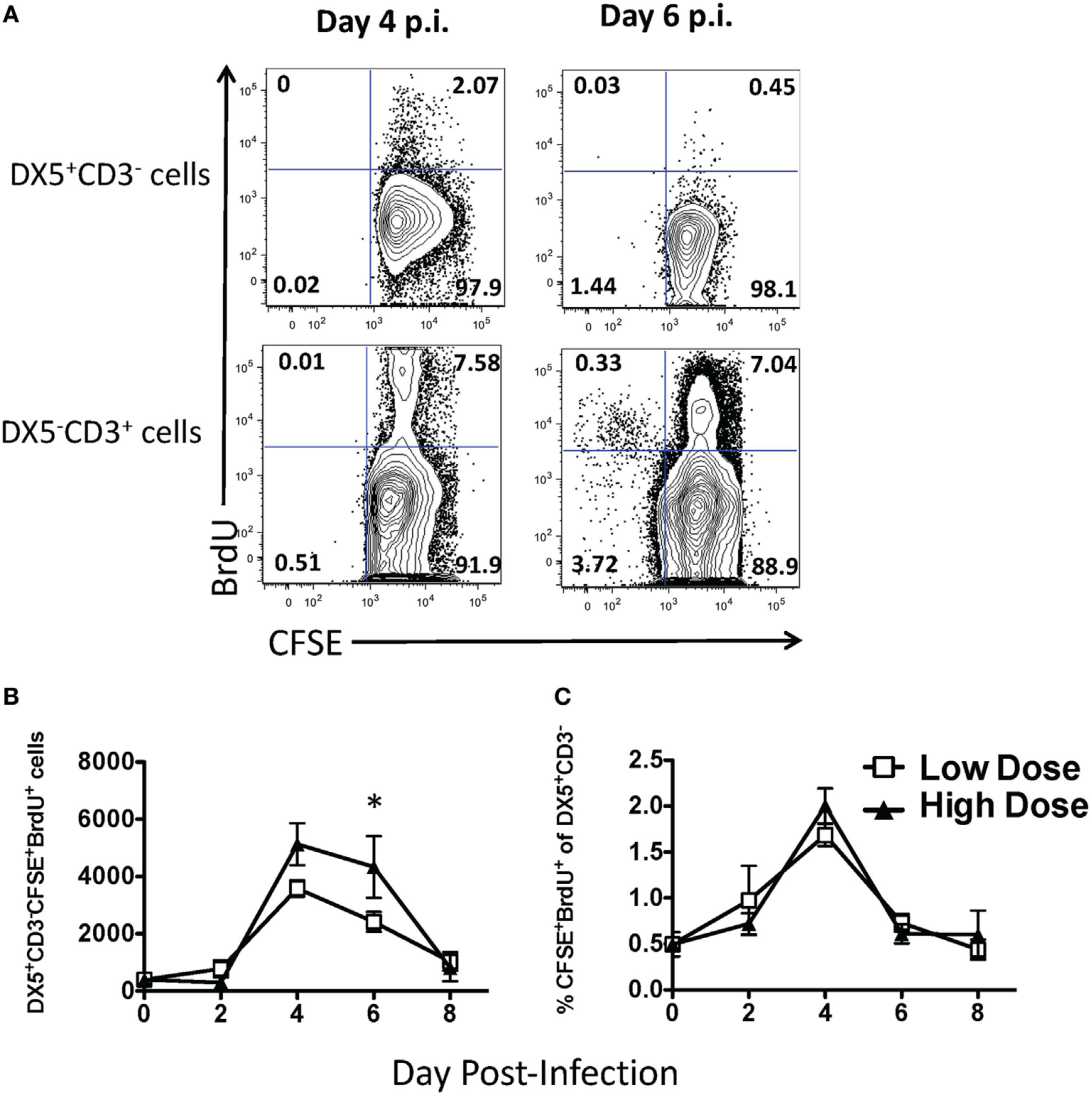
Small frequency of natural killer (NK) cells proliferate in the lung during low- and high-dose influenza A virus (IAV) infections. BALB/c mice were infected with either a high- or low-dose IAV infection and administered carboxyfluorescein succinimidyl ester (CFSE) and bromodeoxyuridine (BrdU) intranasally on the day of analysis, as described in Section “Materials and Methods.” **(A)** Representative flow plots for NK cells (DX5^+^CD3ε^−^ gated) and T cells (DX5^−^CD3ε^+^ gated) in the lungs of day 4 or 6 low-IAV dose-infected mice. The **(B)** number and **(C)** frequency of proliferating NK cells (i.e., the percentage of all DX5^+^CD3ε^−^ cells that were also CFSE^+^BrdU^+^) was determined by flow cytometry. The *p*-values comparing high- vs. low-dose IAV-infected animals are shown. *p < 0.05 (two-tailed unpaired Student’s *t*-test). Data are pooled from three high-dose experiments and two low-dose experiments, with five mice per group, per time point.

Since we observed differential trafficking of NK cells during high- and low-dose IAV infections, we next sought to determine the mechanism(s) regulating this trafficking. The difference in accumulation within the lung between high- and low-dose IAV infections could be due to at least three potential mechanisms: differential levels of (1) apoptosis, (2) proliferation, and/or (3) recruitment. When we examined the level of apoptosis of NK cells *via* active caspase 3/7 and 7-AAD staining, we observed that less than 0.5% of the NK cells were undergoing apoptosis (i.e., caspase 3/7^+^7–AAD^+^) in either the high- or low-dose IAV-infected lungs on day 4 p.i. (not shown). This suggested that differential apoptosis likely does not explain the differing accumulation of NK cells in the lungs after high- and low-dose IAV. Previous studies have examined NK cell proliferation during IAV infection and have shown that the main site of NK cell proliferation is the bone marrow, and only a small percentage of NK cells within the lung incorporate BrdU (33, 34). However, these studies did not directly differentiate between NK cells proliferating within the lung and NK cells proliferating in the periphery before being recruited to the lung. Further, it remains unknown as to what effect the initial dose of IAV has on NK cell proliferation. To quantify NK proliferation specifically within the lung, we used a CFSE followed by BrdU dual-labeling method that we have previously described (27) (Figure 2A). This labeling technique utilizes i.n. CFSE administration to tag cells within the lungs followed by a subsequent BrdU labeling to identify cells that have proliferated within the local lung environment. We observed NK cell proliferation in the lung during both high- and low-dose infections, which peaked at day 4 p.i. When comparing high- to low-IAV infection doses, we observed significantly more NK cells proliferating within the lungs of high-dose-infected mice at day 6 p.i. (Figure 2B). While viral dose affected the overall number of proliferating NK cells, similar frequencies of proliferating NK cells were observed between the two virus doses (Figure 2C), suggesting that the difference observed in the number of proliferating NK cells was reflective of the difference in the total number of NK cells recruited to the lungs, rather than a direct effect of the initial dose of virus on NK cell proliferation itself (Figures 1 and 2C). While our results demonstrate lung-specific NK cell proliferation for the first time, it should be noted that the proportion of NK cells proliferating within the lung is very low when compared to IAV-specific T cell proliferation at these same time points (Figure 2A) (27). At the peak of NK cell proliferation, approximately 2% of NK cells within the lung were found to be CFSE^+^BrdU^+^, while at the same time point, 55–75% of IAV-tetramer^+^ CD8 T cells (27) and 7–8% of all CD3ε^+^ T cells (Figure 2A) within the lung are CFSE^+^BrdU^+^. Finally, although we observed NK cell proliferation within IAV-infected lungs, it is clear that the number of proliferating NK cells and the associated cell expansions could not account for the total increase in NK cell numbers observed within the lung. These data suggest that NK cell recruitment from the periphery into the lung is the main contributor to NK cell accumulation observed in both high- and low-dose IAV-infected lungs.

### NK Cells Are Recruited to the Lung and DLN *via* Chemokine Receptors

**Figure 3:**
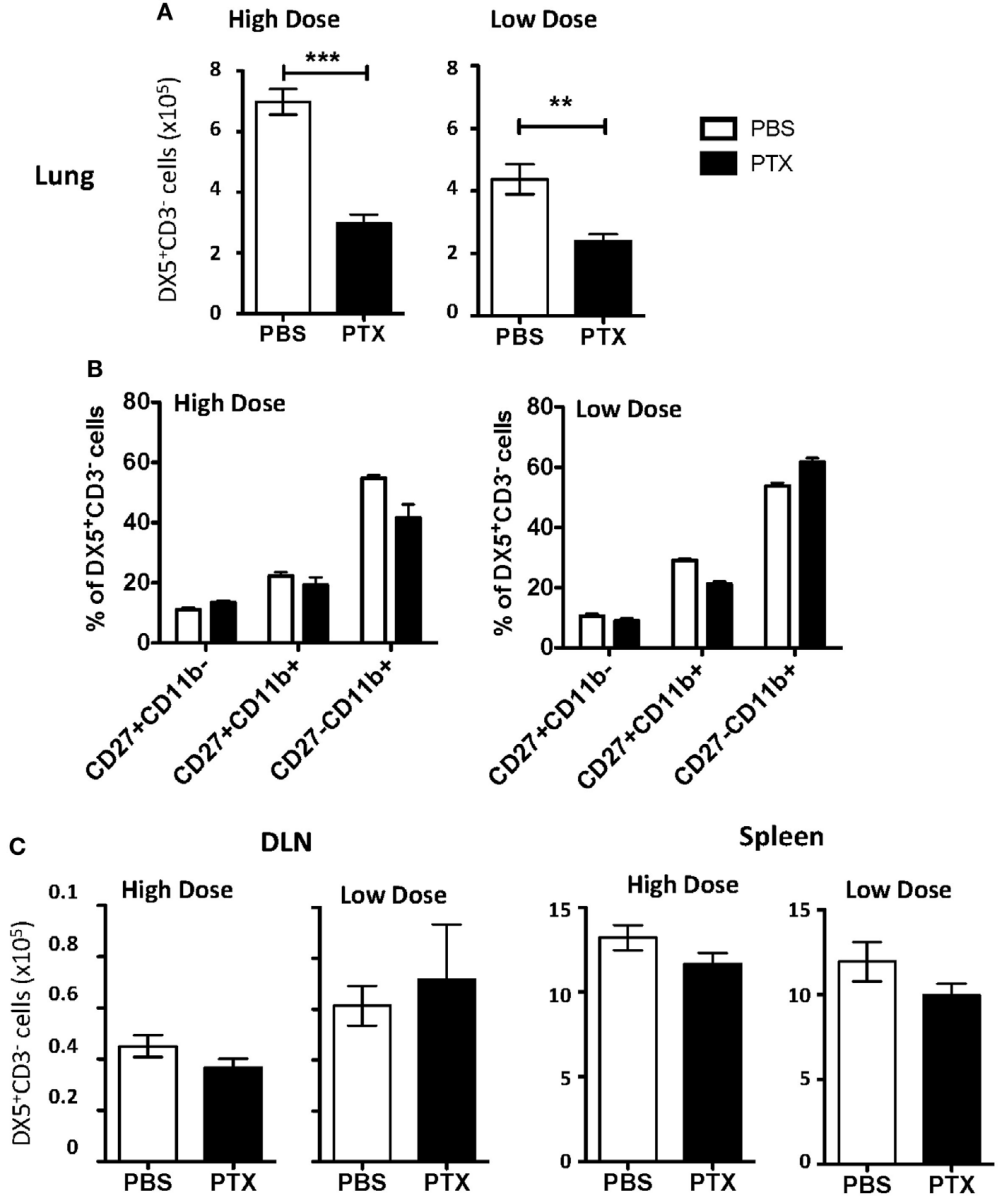
Blockade of chemokine receptor-mediated recruitment results in decreased natural killer (NK) cell accumulation in the influenza A virus (IAV)-infected lung. BALB/c mice were infected with either a high or a low dose of IAV and administered a 500 ng/200 μl intraperitoneal injection of pertussis toxin (PT) or 200 µL phosphate buffered saline (PBS) on days 2, 3, 4, and 5 post infection (p.i.). On day 6 p.i., lungs **(A)**, draining lymph node (DLN) (per node) **(C)**, and spleens **(C)** were harvested from high-dose-infected mice or low-dose-infected mice, and the number and frequency of NK cells (DX5^+^CD3ε^−^ gated) were determined by flow cytometry. **(B)** NK cells (DX5^+^CD3ε^−^ gated) in the lung were further characterized by CD27 and CD11b expression to determine changes in functional NK subset distribution. Shown are the *p*-values comparing PT-treated mice to PBS-treated mice. **p < 0.005, ***p < 0.001 (two-tailed unpaired Student’s *t*-test). Data are pooled from three high-dose experiments and two low-dose experiments, with five mice per group.

As NK cell proliferation within the lung could not account for the total NK cell accumulation observed, we next determined if NK cells were recruited to the lung during IAV infection *via* chemokine receptors. Chemokine receptors are G protein-coupled receptors whose activation is required for gradient-directed recruitment of cells into tissues. To determine if chemokine receptors were necessary for NK cell accumulation within the IAV-infected lung, chemokine receptor activation was blocked by administering PT, an inhibitor of G protein-coupled receptors (35), to mice infected with either a high or a low dose of IAV. PT significantly decreased the number of NK cells in the lungs of both groups of IAV-infected mice (Figure 3A). Further, blocking chemokine receptor activation did not selectively inhibit the recruitment of a specific subset of NK cells, as there was no change in the subset distribution between PBS- and PT-treated mice (Figure 3B). Notably, while PBS-treated mice infected with a high dose of IAV had significantly more NK cells in the lungs at day 6 p.i. than low-dose-infected mice (Figures 1 and 3A), there was no difference in the number of NK cells between high- and low-dose infections in PT-treated mice, suggesting that the difference in NK cell accumulation in the lung between high- and low-dose infections in non-PT treated mice is likely due to enhanced recruitment *via* chemokine receptors. No difference was observed in NK cell numbers within the DLN or spleens between PT- and PBS-treated mice, suggesting that the difference in NK cell accumulation observed within the lung was due to a decreased trafficking, rather than a toxic effect of PT treatment (Figure 3C).

**Figure 4:**
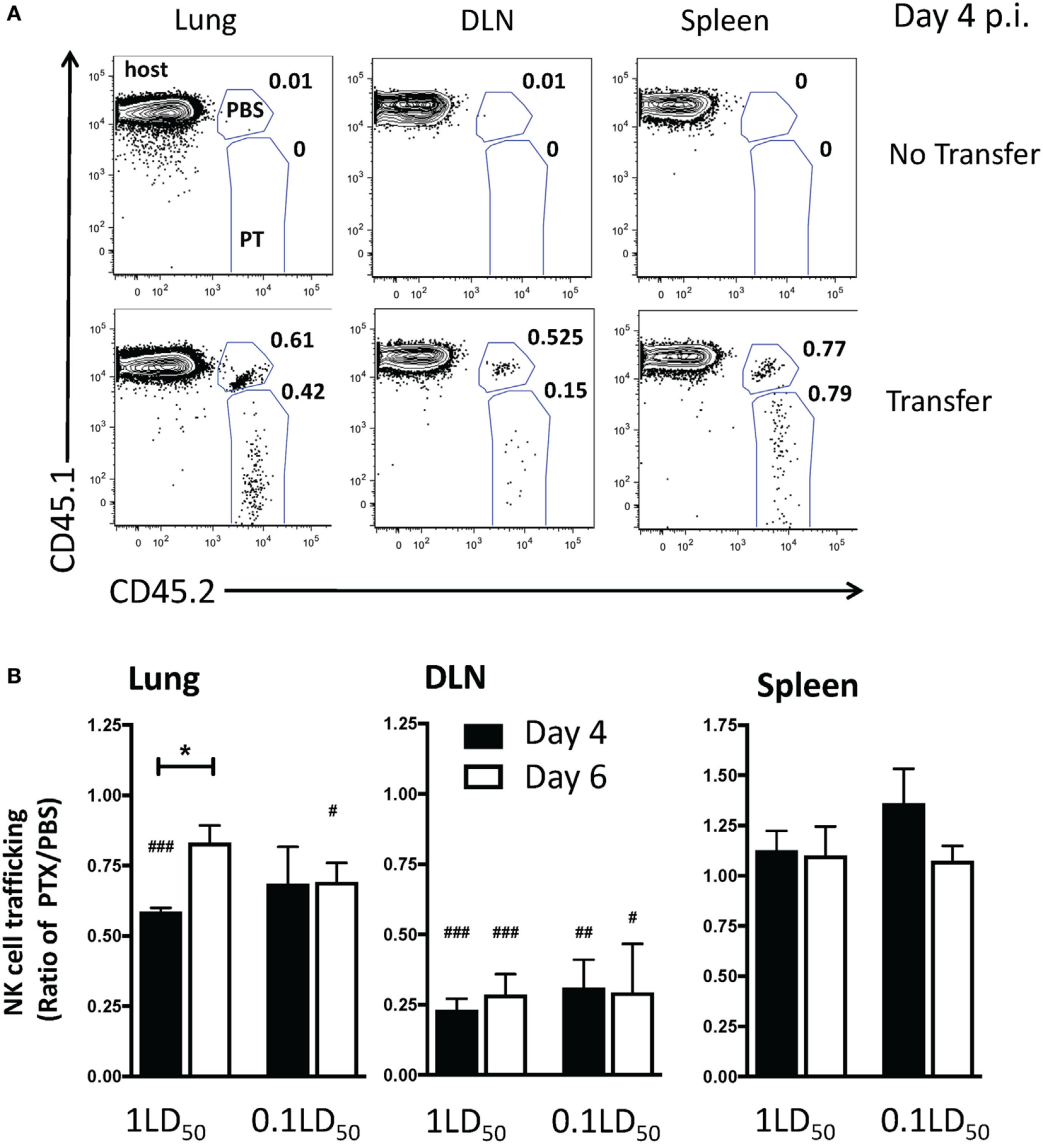
Inhibition of chemokine receptor signaling in natural killer (NK) cells results in a decreased accumulation in the lungs and draining lymph nodes (DLNs) of influenza A virus (IAV)-infected mice. C57Bl/6 (CD45.1) host mice were infected with a high- or low-dose of IAV. 1 × 10^5^ donor C57Bl/6 NK cells treated with pertussis toxin (PT) (CD45.2^+^) and 1 × 10^5^ C57Bl/6 NK cells treated with phosphate buffered saline (PBS) (CD45.1^+^CD45.2^+^) were transferred into infected host mice for 16–18 h before analysis (i.e., transferred on day 3 p.i. for day 4 analysis, or day 5 p.i. for day 6 analysis). A group of non-NK cell transferred mice was used as a control. **(A)** Representative flow plots for NK cells (NK1.1^+^CD3ε^−^) on day 4 p.i. in high-dose IAV lungs, DLN, and spleen showing host (CD45.1^+^CD45.2^−^), PBS-treated donor (CD45.1^+^CD45.2^+^), and PT-treated donor (CD45.1^−^CD45.2^+^) NK cells. **(B)** the normalized ratio of PT-treated NK cells to PBS-treated control NK cells in the lungs, DLN, and spleen of infected recipients is shown. Note: equal trafficking between PBS- and PT-treated NK cells = 1, an increased PT-treated NK cell trafficking >1, and a decreased PT-treated NK cell trafficking <1. *p*-values comparing PT-treated NK cell trafficking with PBS-treated cells within the same group are shown. ^#^*p* < 0.05, ^###^*p* < 0.001 (two-tailed paired Student’s *t*-test). *p < 0.05 represents comparison of the PT/PBS ratios between the indicated groups. High- and low-dose experiments were performed once with four mice per group.

To determine a more specific role for chemokine receptors on NK cells in NK cell trafficking during IAV infection, NK cells from CD45 congenic mice were incubated with PT (CD45.1^−^CD45.2^+^) or PBS (CD45.1^+^CD45.2^+^) prior to adoptive transfer into IAV-infected hosts (CD45.1^+^CD45.2^−^) (Figure 4A). We observed a significant reduction in PT-treated NK cell trafficking to the lung compared to PBS-treated donor NK cells in high-dose IAV-infected mice at day 4 p.i. and in low-dose IAV-infected mice at day 6 p.i. (i.e., lower numerical ratio as a result of a reduced recruitment of PT-treated donor cells compared to PBS-treated NK cells, Figure 4B). In contrast to our findings *in vivo* in PT-treated mice, *in vitro* PT-treated NK cell trafficking to the DLN was significantly reduced compared to PBS-treated NK cells in both high- and low-dose IAV-infected mice on days 4 and 6 p.i. (Figure 4B). The reduced trafficking of PT-treated NK cells into the DLN at day 6 p.i. (Figure 4B) suggests that while the overall number of NK cells in the DLN is decreasing at that time, there is still a detectable influx of NK cells and that trafficking into the DLN at later time points is mediated by chemokine receptors. No difference in NK cell trafficking to the spleen was observed (Figure 4B). These data further support a role for chemokine receptor-mediated trafficking of NK cells to the lungs and DLN during IAV infection.

### CXCR3 Expression on NK Cells Increases NK Cell Recruitment to the Lungs and DLN of IAV-Infected Mice

**Figure 5:**
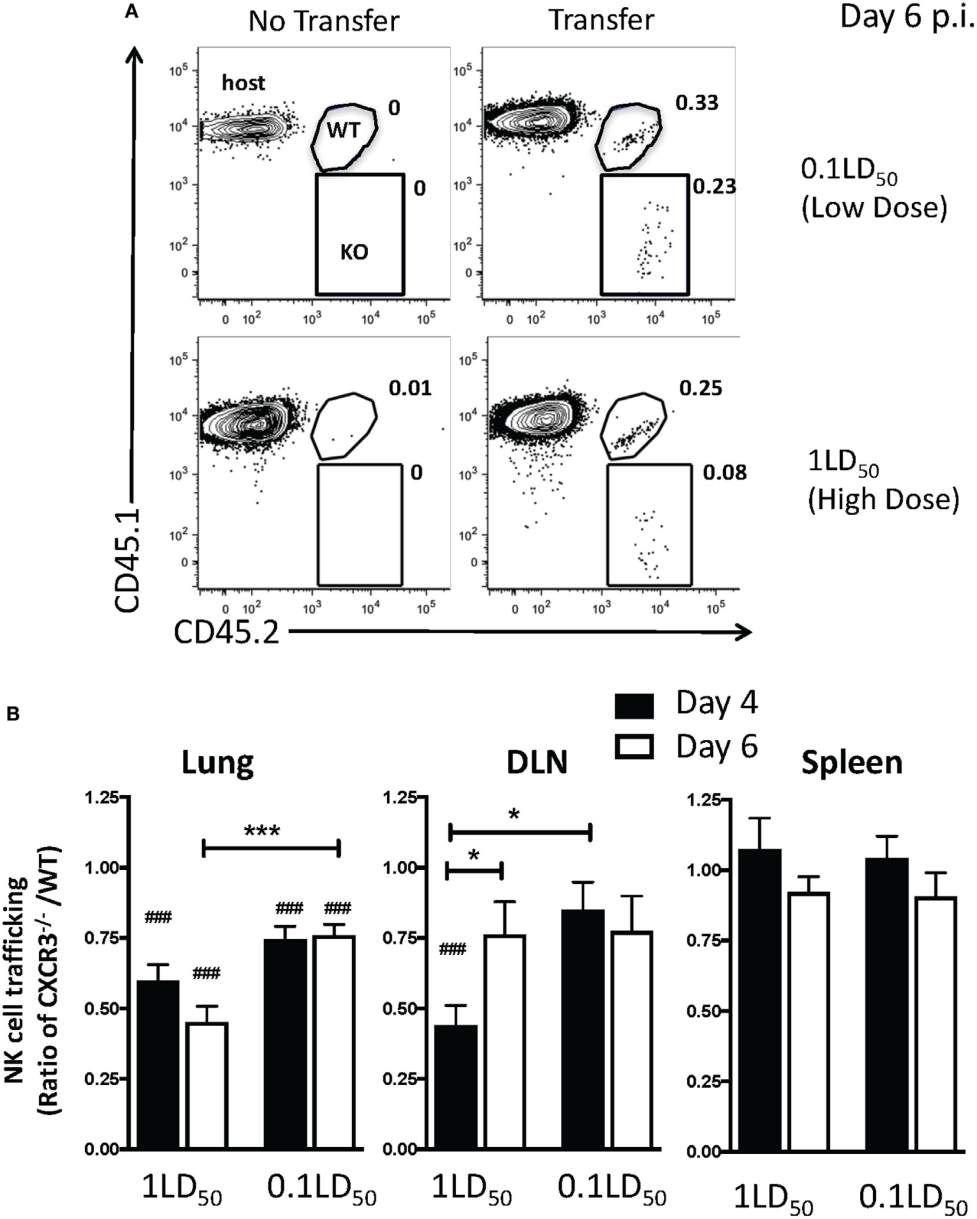
CXCR3 expression by natural killer (NK) cells increases NK cell trafficking to the lung and draining lymph node (DLN) during influenza A virus (IAV) infection. C57Bl/6 (CD45.1) host mice were infected with a high or a low dose of IAV. 5 × 10^5^ donor WT C57Bl/6 (CD45.1/CD45.2) and 5 × 10^5^ CXCR3^−/−^ (CD45.2) NK cells were transferred into infected host mice for 16–18 h before analysis. **(A)** Representative flow plots for NK cells (NK1.1^+^CD3ε^−^) on day 6 post infection (p.i.) in high- or low-dose IAV lungs showing host (CD45.1^+^CD45.2^−^), WT donor (CD45.1^+^CD45.2^+^), and CXCR3^−/−^ donor (CD45.1^−^CD45.2^+^) NK cells. **(B)** The normalized ratio of CXCR3^−/−^ NK cells to WT NK cells in the lungs, DLN, and spleen of infected recipients is shown. Note: equal trafficking between WT and CXCR3^−/−^ NK cells = 1. *p*-values comparing WT to CXCR3^−/−^ NK cell trafficking (two-tailed paired Student’s *t*-test) within the same group are shown. ^#^*p* < 0.05, ^###^*p* < 0.001, and comparing, as indicated, the CXCR3^−/−^ NK cells:WT NK cell ratio between high- and low-dose IAV-infected mice or by time post infection. *p < 0.05, ***p < 0.001. Data are pooled from two high-dose experiments and three low-dose experiments, with five mice per group.

**Figure 6:**
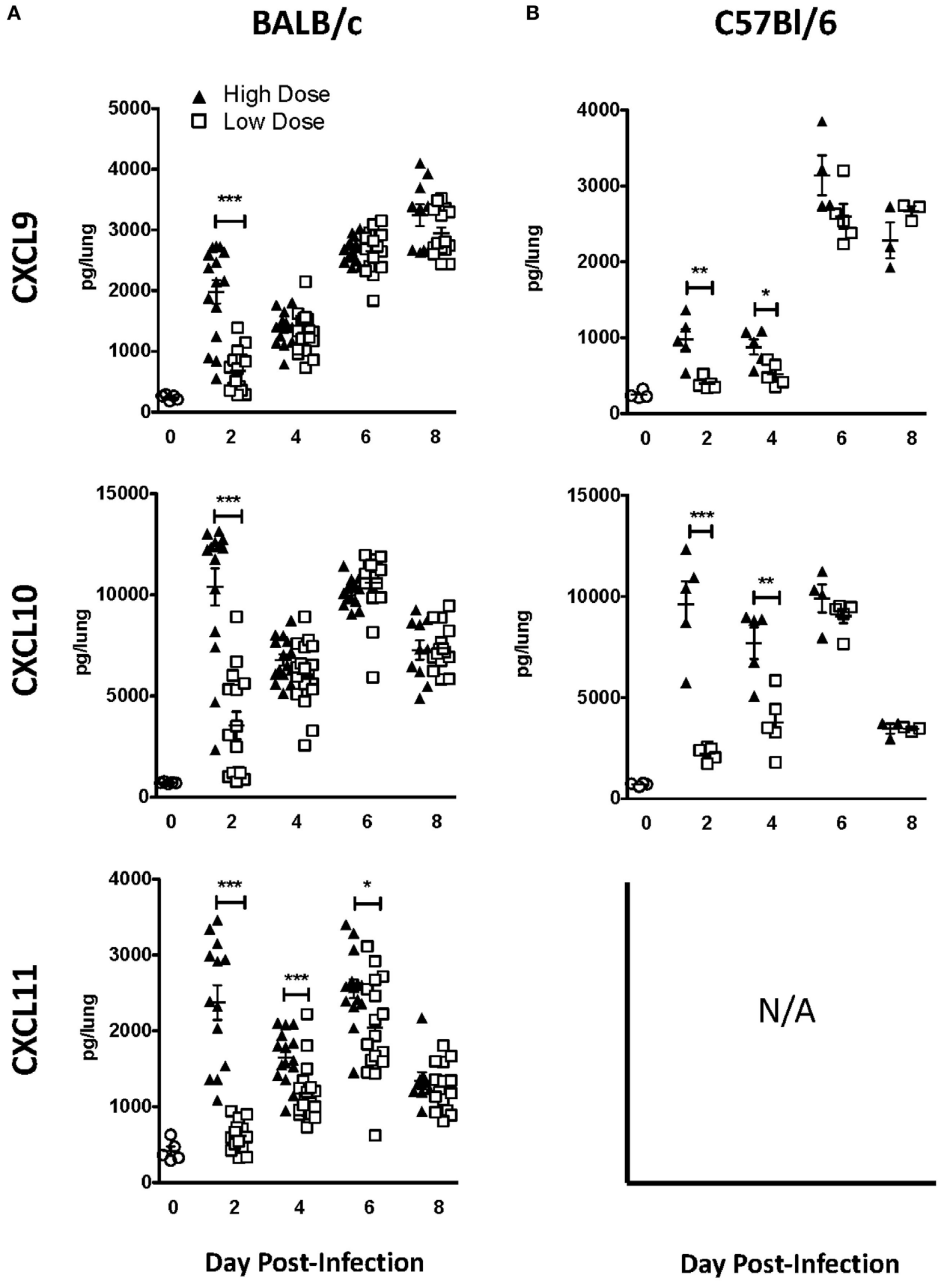
CXCR3 ligands are upregulated in the lungs during influenza A virus (IAV) infection and expressed at higher levels in the lungs of high-dose-infected mice. **(A)** BALB/c and **(B)** C57Bl/6 mice were infected with either a high- or low-dose IAV. Lungs were homogenized, and the levels of CXCL9, CXCL10, and CXCL11 [not expressed (N/A) in C57Bl/6 mice] were quantified by ELISA. *p*-values comparing chemokine levels in high- vs. low-dose IAV-infected mice are shown. *p < 0.05, **p < 0.005, ***p < 0.001 (two-tailed unpaired Student’s *t*-test). BALB/c data are pooled from two separate experiments with at least five mice per group. C57Bl/6 data are representative of one experiment with at least three mice per group.

Since our results implicate chemokine receptors in NK cell accumulation in the lung, we determined which chemokine receptors were used for NK cell trafficking to the IAV-infected lung. CXCR3 is an important chemokine receptor for NK cell homing in homeostasis (20), and the receptor is used by CD8 T cells to migrate into the IAV-infected lung (23). Therefore, to determine if CXCR3 was necessary for NK cell recruitment to the IAV-infected lung, we again utilized a competitive trafficking assay. Donor congenic WT CXCR3^+/+^ (CD45.1^+^CD45.2^+^) and CXCR3^−/−^ (CD45.1^−^CD45.2^+^) NK cells were transferred into IAV-infected CXCR3^+/+^ hosts (CD45.1^+^CD45.2^−^) (Figure 5A). There was a significant decrease in CXCR3^−/−^ NK cell trafficking compared to donor WT NK cell trafficking (i.e., KO:WT ratio <1) to the lung and DLN in high-dose-infected mice and to the lungs of low-dose-infected mice (Figure 5B). Notably, the ratio of recruitment of donor CXCR3^−/−^ to CXCR3^+/+^ NK cell was also significantly reduced in high-dose compared to low-dose IAV-infected mice at day 6 p.i. (Figure 5B), suggesting that CXCR3 expression on NK cells could be more important for recruitment to the lung in high-dose IAV infections. Therefore, the amount of CXCR3 ligands, CXCL9, CXCL10, and CXCL11, was quantified in the lungs of high- and low-dose-infected mice (Figure 6) to determine if differences in ligand expression correlated with the differences observed in the competitive trafficking assay (Figure 5B). All CXCR3 ligands were significantly increased in the lungs of high-dose compared to low-dose-infected BALB/c mice at day 2 p.i., while only CXCL11 was expressed in higher amounts from days 2 to 6 p.i. (Figure 6A). Similarly, CXCL9 and CXCL10 were significantly greater in the lungs of high-dose vs. low-dose-infected C57Bl/6 mice at both days 2 and 4 p.i. (Figure 6B). The increased amount of CXCR3 ligand found in the lungs of mice receiving a high-dose of IAV correlated with the reduced CXCR3^−/−^ NK cell trafficking that was observed in high-dose IAV-infected mice (Figure 5B).

### CCR5 Localizes NK Cells to IAV-Infected Epithelium

**Figure 7:**
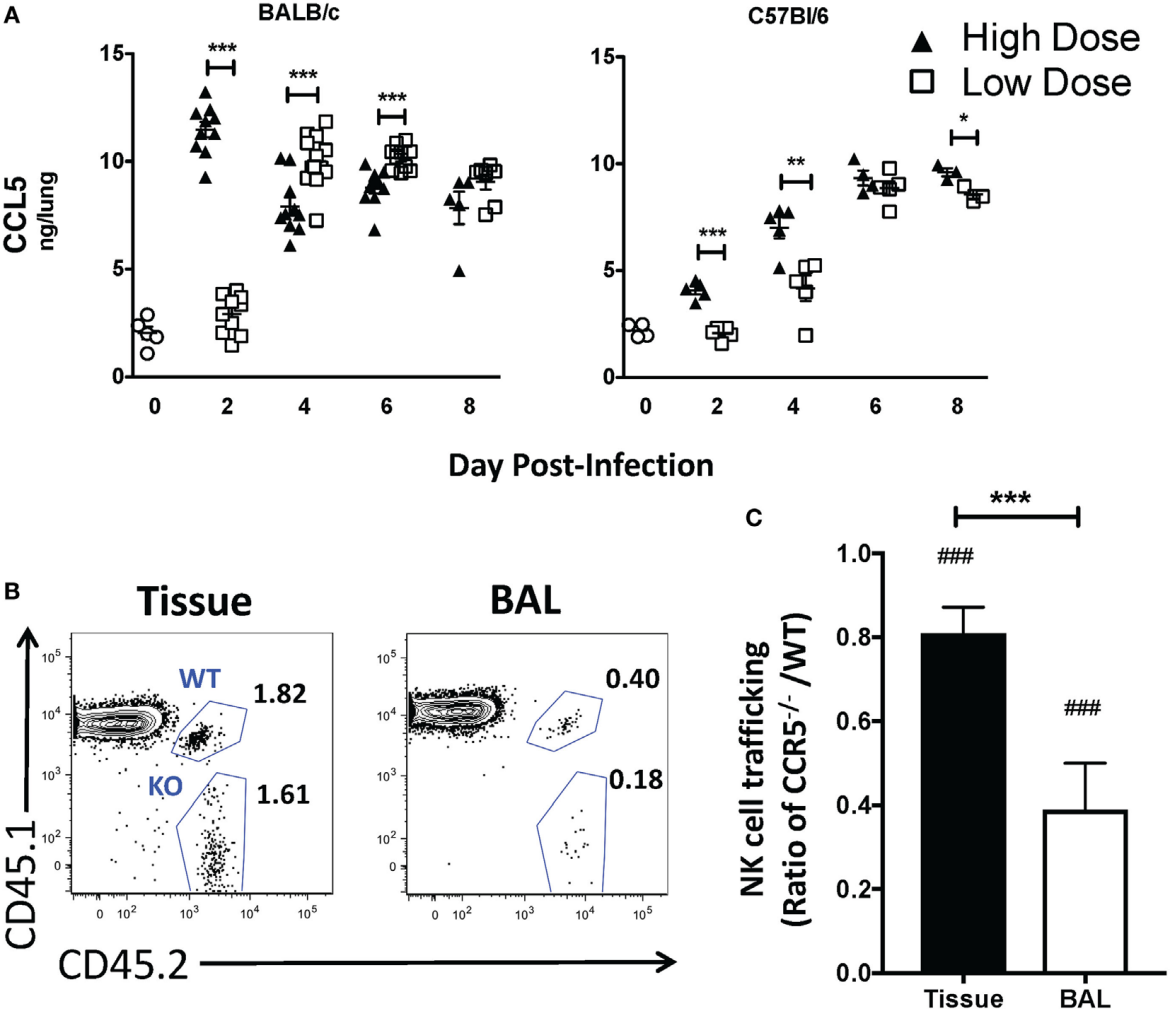
CCL5 is upregulated in the lungs during high- and low-dose influenza A virus (IAV) infections. **(A)** BALB/c (left) and C57Bl/6 (right) mice were infected with high- and low-dose A/PR/8/34. Lungs were harvested at indicated time points, and CCL5 in supernatants was quantified by ELISA. Uninfected mice were used as naïve controls. *p*-values comparing CCL5 levels in high- vs. low-dose IAV-infected mice are shown. Data were obtained from samples as in Figure 6. **(B,C)** C57Bl/6 (CD45.1) host mice were infected with a high dose of IAV. 3 × 10^5^ donor WT C57Bl/6 (CD45.1/CD45.2) and 3 × 10^5^ CCR5^−/−^ (CD45.2) natural killer (NK) cells were transferred into infected host mice for 16–18 h before analysis. **(B)** Representative flow plots for NK cells (NK1.1^+^CD3ε^−^) in the bronchoalveolar lavage (BAL) vs. lung tissue showing host (CD45.1^+^CD45.2^−^), WT donor (CD45.1^+^CD45.2^+^), and CCR5^−/−^ donor (CD45.1^−^CD45.2^+^) NK cells. **(C)** Shown is the normalized ratio of CCR5^−/−^ NK cells to WT NK cells in the lungs or BAL fluid of infected recipients. Data are from one experiment with six mice per group. Note: equal trafficking between WT and CCR5^−/−^ NK cells = 1. *p*-values comparing WT to CCR5^−/−^ NK cell trafficking (two-tailed paired Student’s *t*-test) within the same group are shown. ^###^*p* < 0.001 and comparing the ratio of CCR5^−/−^/WT NK cell trafficking in the BAL vs. lung tissue. ***p < 0.001.

While our above studies and others suggest that CXCR3 aids in NK cell recruitment to both the naïve- and IAV-infected lung (20), it is not known if or how NK cells are localized to infected epithelial cells. Similar to NK cells, CXCR3 expression on CD8 T cells increases their recruitment to the lung during IAV infection (23). However, once in the lungs, CCR5 is required for memory CD8 T cell localization to the IAV-infected airway (22). Ligands for CCR5 (CCL3, CCL4, and CCL5) are upregulated in the lung of both BALB/c and C57Bl/6 mice [Figure 7A (36, 37)]. Interestingly, while similar trends in CXCR3 ligands were observed between BALB/c and C57Bl/6 mice, the pattern of relative CCL5 expression appeared to be inverted at later time points. While CCL5 was significantly upregulated at day 2 in high-dose IAV-infected lungs in both strains, CCL5 continued to have a higher expression in high-dose IAV-infected lungs in C57Bl/6 mice, while CCL5 expression was significantly higher in low-dose IAV-infected BALB/c mice (Figure 7A). Despite the difference in expression pattern, CCL5 was significantly upregulated in the lungs compared to naïve mice on days 2–8 during high-dose IAV infection and on days 4–8 during low-dose IAV infections for both mouse strains (Figure 7A, *p* < 0.05, student’s unpaired *t*-test). Therefore, we hypothesized that CCR5 expression on NK cells would be required for localization to the infected epithelium. Previously, it had been shown that IAV-infected CCR5^−/−^ mice have less NK cell accumulation in the lung than IAV-infected WT mice (15). However, this defect in NK cell recruitment could be due to a requirement for CCR5 in NK cell recruitment to the IAV-infected lung, or due to the known proliferative defects of CCR5^−/−^ NK cells, which results in decreased NK cell numbers in the periphery of naïve mice (15). To more directly test if CCR5 was required for NK cell accumulation in the IAV-infected lung, CCR5^−/−^ NK cells (CD45.1^−^CD45.2^+^) and WT NK cells (CD45.1^+^CD45.2^+^) were adoptively transferred into high-dose IAV-infected WT C57Bl/6 recipients (CD45.1^+^CD45.2^−^) (Figure 7B). A significant decrease in the accumulation of CCR5^−/−^ NK cells compared to WT NK cells in the lung tissue (i.e., ratio <1, Figure 7C) was observed at day 6 p.i. To further determine if CCR5 was used for NK cell localization within the IAV-infected lungs, CCR5^−/−^ and WT NK cell accumulation within the bronchoalveolar lavage fluid was determined. The ability of CCR5^−/−^ NK cells to traffic to the IAV-infected airway was significantly reduced compared to WT NK cells (Figure 7C), and the ability of CCR5^−/−^ NK cells to traffic to the airway was significantly reduced compared to lung tissue, suggesting that while CCR5 may aid in NK cell recruitment to the lung tissue, it is also important for NK cell localization to the infected epithelium.

## Discussion

Our results show that NK cells are actively recruited to the lung and lung DLN during IAV infection, consistent with previous studies (5). However, herein, we show preferential organ-specific recruitment (31, 38, 39) that is dependent on the dose of infection. Recruitment to the lung is increased in high-dose IAV infections, and accumulation of NK cells within the DLN is increased in low-dose IAV infections. Importantly, studies have shown disparate roles for NK cells in immunity against IAV, with NK cells playing a protective role in lower-dose IAV infections and contributing to lethal pathology in lethal dose infections (5, 8, 9, 12, 13). While the results presented herein argue for differential NK cell recruitment and trafficking based on the IAV infection dose, they also suggest that such differences may not alter pulmonary virus titers from days 2 to 8 p.i. Furthermore, our results show that the loss of NK cells prior to both high- and low-dose IAV infections does not alter IAV morbidity or mortality. However, at this time, it is difficult to interpret the direct contribution of NK cells to the overall virus control and pathology as compensatory changes in other immune cell populations that contribute to these aspects occur in the absence of NK cells or during different infection doses. For example, our preliminary studies suggest that IAV-specific CD4 T cell responses may be increased in the absence of NK cells (not shown). Furthermore, our prior studies have demonstrated reduced levels of IAV-specific CD8 T cells in the lungs of 1LD_50_ dose vs. 0.1 LD_50_ dose IAV-infected mice (26). Therefore, future studies determining if the magnitude of the NK cell response within the lungs or DLN is a significant contributor to pathology or virus control in both high- and low-dose IAV infections will also need to assess the modulation of other immune cell populations critical to IAV immunity and pathology at the same time.

Herein, we show for the first time that there is a lung-specific NK cell proliferation during IAV infection by labeling pulmonary cells with CFSE in addition to intranasal BrdU administration. Our results are consistent with other studies that administered BrdU systemically (33, 34) and *in vitro* NK cell: alveolar macrophage co-cultures (10). Further, we show that NK cell proliferation within the lung is not dependent on IAV virus titers, as the same frequency of NK cells proliferated within the lung during both high- and low-dose IAV infections. Our findings that NK cell proliferation within the lung does not account for the total accumulation of NK cells and that PT treatment decreases NK cell accumulation within the lung suggest that NK cell accumulation in the lung is largely due to a directed recruitment rather than a local proliferation.

CXCR3 is important for NK cell trafficking to the lung both in homeostasis and in bacterial infection (20, 21). Herein, we show that CXCR3 also plays a role in recruiting NK cells to the lung during IAV infection, as CXCR3^−/−^ NK cells did not traffic to the lung as efficiently as WT NK cells. The difference between WT and CXCR3^−/−^ NK cell trafficking was more pronounced in mice infected with a high-dose IAV infection, and CXCR3 ligands were significantly higher in the lungs of high-dose compared to low-dose IAV-infected mice. Whether CXCR3 plays a more dominant role in NK cell recruitment during high-dose IAV infections or is solely responsible for the increased accumulation of NK cells within high-dose IAV-infected lungs remains to be determined. Importantly, although CXCR3^−/−^ NK cells had significantly reduced recruitment compared to CXCR3^+/+^ NK cells, CXCR3^−/−^ NK cells did accumulate in IAV-infected lungs. This suggests that the recruitment of NK cells to the lung during IAV infection may be multifactorial, most likely utilizing several chemokine receptors. Indeed, we have shown herein that CCR5 is also used for NK cell recruitment to the IAV-infected lung (Figure 7C). CCR5^−/−^ NK cells had impaired accumulation to the IAV-infected lung compared to WT NK cells; however, the decrease in CCR5^−/−^ NK cell accumulation was not as significant as the decrease in CXCR3^−/−^ NK cells accumulation when compared to WT NK cells (Figures 5B and 7C). In addition to CXCR3 and CCR5, CCR2 is important for NK cell trafficking during IAV infection. While others have shown that CCR2 on NK cells does not affect accumulation within the IAV-infected lung tissue, it is used by NK cells to traffic to the IAV-infected airways (19). It is possible that CCR2, among other chemokine receptors, is able to compensate for the loss of CXCR3 or CCR5 in NK cell migration to the IAV-infected lung.

Our experiments utilizing PT treatment to inhibit NK cell trafficking resulted in opposing findings within the DLN, where NK cell accumulation was not affected in PT-treated mice, but was significantly inhibited when NK cells were treated with PT *in vitro*. Chemokine receptor signaling is required for both cell recruitment to organs and cell egress from organs. CXCR3 has been shown to increase NK cell recruitment to inflammation DLNs, while S1P5 and homeostatic chemokines are utilized for NK cell egress from lymph nodes (31, 32, 40, 41). By treating mice with PT for 4 days *in vivo* prior to analysis, we likely uncoupled these two processes, and it is not possible to determine if the lack of difference observed in NK cell numbers in the DLN between *in vivo* PT and PBS-treated mice is due to changes in recruitment to or egress from the lymph node. As the adoptive transfer model examines NK cell recruitment for 16–18 h after transfer, we are more likely to be observing NK cell recruitment to the DLN, rather than recruitment and egress. While the number of NK cells in the DLN begins to decrease by day 6 p.i. (Figure 1B), NK cell recruitment to the DLN could still be occurring at day 5 p.i., when PBS- and PT-treated NK cells were adoptively transferred to IAV-infected hosts. Finally, NK cells in *in vivo* PT- and PBS-treated mice had been exposed to inflammation for 5 days prior to analysis, while *in vitro* PBS- and PT-treated NK cells were adoptively transferred into IAV-infected hosts and exposed to inflammation for 16–18 h before analysis. It is possible that this difference in inflammation exposure results in temporal differences in NK cell trafficking. Curiously, a recent study has also evaluated the role of CXCR3 in the trafficking of NK cells to the DLN during influenza infection and found that despite the expression of CXCR3 predominantly on the immature CD27^+^CD11b^−^ subset, the accumulation of CXCR3^−/−^ NK cells in the DLN was largely unaffected (31). This result is consistent with our observations during low-dose IAV infection (Figure 5). However, during high-dose infection, our results suggest a greater importance for CXCR3 in NK cell recruitment to the DLN, as CXCR3^−/−^ NK cell recruitment was reduced (Figure 5B). Altogether, these results suggest that a multifactorial model of chemokine receptor directed recruitment into the DLN where CXCR3 may play a more prominent role during high inflammatory or high pathogenic infection conditions.

Overall, our data suggest that the severity of IAV infection dictates the localization and number of NK cells in lymphoid tissue and the site of infection and that this localization is partially dependent on CXCR3 and CCR5 and differential expression of CXCR3 and CCR5 ligands.

## Ethics Statement

All procedures were approved by the University of Iowa Animal Care and Use Committee.

## Author Contributions

LC and EH conducted the CCR5 adoptive transfer experiments. LC conducted the trafficking kinetics, BrdU, PTX, CXCR3 adoptive transfer, and ELISA experiments, and these experiments are part of her dissertation (42). ZZ conducted the viral titer experiments. LC, EH, JH, ZZ, and KL contributed to data analysis and interpretation. JH and KL contributed to the conception of the work. LC and EH wrote the manuscript with substantial input from JH and KL. All the authors approved the final version of the manuscript.

## Conflict of Interest Statement

The authors declare that the research was conducted in the absence of any personal, professional, or financial conflicts of interest.

## Abbreviations

BAL, bronchoalveolar lavage; BrdU, bromodeoxyuridine; CFSE, carboxyfluorescein succinimidyl ester; DLN, draining lymph node; IAV, influenza A virus; mAb, monoclonal antibody; NK, natural killer; PBS, phosphate buffered saline; p.i., post-infection; PT, pertussis toxin.

## References

[1] Colonna M Jonjic S Watzl C. Natural killer cells: fighting viruses and much more. Nat Immunol (2011) 12(2):107–10. 10.1038/ni0211-107 21245897

[2] Biron CA Byron KS Sullivan JL Severe herpesvirus infections in an adolescent without natural killer cells. N Engl J Med (1989) 320(26):1731–5. 10.1056/NEJM198906293202605 2543925

[3] Burshtyn DN NK cells and poxvirus infection. Front Immunol (2013) 4:7 10.3389/fimmu.2013.00007 23372568 PMC3556567

[4] Du N Zhou J Lin X Zhang Y Yang X Wang Y Differential activation of NK cells by influenza A pseudotype H5N1 and 1918 and 2009 pandemic H1N1 viruses. J Virol (2010) 84(15):7822–31. 10.1128/JVI.00069-10 20484512 PMC2897595

[5] Gazit R Gruda R Elboim M Arnon TI Katz G Achdout H Lethal influenza infection in the absence of the natural killer cell receptor gene Ncr1. Nat Immunol (2006) 7(5):517–23. 10.1038/ni1322 16565719

[6] Ge MQ Ho AW Tang Y Wong KH Chua BY Gasser S NK cells regulate CD8+ T cell priming and dendritic cell migration during influenza A infection by IFN-gamma and perforin-dependent mechanisms. J Immunol (2012) 189(5):2099–109. 10.4049/jimmunol.1103474 22869906

[7] Kos FJ Engleman EG. Role of natural killer cells in the generation of influenza virus-specific cytotoxic T cells. Cell Immunol (1996) 173(1):1–6. 10.1006/cimm.1996.0245 8871595

[8] Stein-Streilein J Guffee J. *In vivo* treatment of mice and hamsters with antibodies to asialo GM1 increases morbidity and mortality to pulmonary influenza infection. J Immunol (1986) 136(4):1435–41. 3944461

[9] Zhou K Wang J Li A Zhao W Wang D Zhang W Swift and strong NK cell responses protect 129 mice against high-dose influenza virus infection. J Immunol (2016) 196(4):1842–54. 10.4049/jimmunol.1501486 26773146

[10] Wang Y Li T Chen Y Wei H Sun R Tian Z. Involvement of NK cells in IL-28B-mediated immunity against influenza virus infection. J Immunol (2017) 199(3):1012–20. 10.4049/jimmunol.1601430 28637903

[11] Ishikawa H Ino S Sasaki H Fukui T Kohda C Tanaka K. The protective effects of intranasal administration of IL-12 given before influenza virus infection and the negative effects of IL-12 treatment given after viral infection. J Med Virol (2016) 88(9):1487–96. 10.1002/jmv.24494 26864280

[12] Abdul-Careem MF Mian MF Yue G Gillgrass A Chenoweth MJ Barra NG Critical role of natural killer cells in lung immunopathology during influenza infection in mice. J Infect Dis (2012) 206(2):167–77. 10.1093/infdis/jis340 22561366

[13] Zhou G Juang SW Kane KP. NK cells exacerbate the pathology of influenza virus infection in mice. Eur J Immunol (2013) 43(4):929–38. 10.1002/eji.201242620 23436540

[14] Fang M Roscoe F Sigal LJ. Age-dependent susceptibility to a viral disease due to decreased natural killer cell numbers and trafficking. J Exp Med (2010) 207(11):2369–81. 10.1084/jem.20100282 20876312 PMC2964566

[15] Khan IA Thomas SY Moretto MM Lee FS Islam SA Combe C CCR5 is essential for NK cell trafficking and host survival following *Toxoplasma gondii* infection. PLoS Pathog (2006) 2(6):e49. 10.1371/journal.ppat.0020049 16789839 PMC1475660

[16] Salazar-Mather TP Orange JS Biron CA. Early murine cytomegalovirus (MCMV) infection induces liver natural killer (NK) cell inflammation and protection through macrophage inflammatory protein 1alpha (MIP-1alpha)-dependent pathways. J Exp Med (1998) 187(1):1–14. 10.1084/jem.187.1.1 9419206 PMC2199190

[17] Gregoire C Chasson L Luci C Tomasello E Geissmann F Vivier E The trafficking of natural killer cells. Immunol Rev (2007) 220:169–82. 10.1111/j.1600-065X.2007.00563.x 17979846 PMC7165697

[18] Morrison BE Park SJ Mooney JM Mehrad B. Chemokine-mediated recruitment of NK cells is a critical host defense mechanism in invasive aspergillosis. J Clin Invest (2003) 112(12):1862–70. 10.1172/JCI18125 14679181 PMC296992

[19] van Helden MJ Zaiss DM Sijts AJ. CCR2 defines a distinct population of NK cells and mediates their migration during influenza virus infection in mice. PLoS One (2012) 7(12):e52027. 10.1371/journal.pone.0052027 23272202 PMC3521727

[20] Jiang D Liang J Hodge J Lu B Zhu Z Yu S Regulation of pulmonary fibrosis by chemokine receptor CXCR3. J Clin Invest (2004) 114(2):291–9. 10.1172/JCI16861 15254596 PMC449741

[21] Widney DP Hu Y Foreman-Wykert AK Bui KC Nguyen TT Lu B CXCR3 and its ligands participate in the host response to *Bordetella bronchiseptica* infection of the mouse respiratory tract but are not required for clearance of bacteria from the lung. Infect Immun (2005) 73(1):485–93. 10.1128/IAI.73.1.485-493.2005 15618188 PMC538932

[22] Kohlmeier JE Miller SC Smith J Lu B Gerard C Cookenham T The chemokine receptor CCR5 plays a key role in the early memory CD8+ T cell response to respiratory virus infections. Immunity (2008) 29(1):101–13. 10.1016/j.immuni.2008.05.011 18617426 PMC2519120

[23] Fadel SA Bromley SK Medoff BD Luster AD. CXCR3-deficiency protects influenza-infected CCR5-deficient mice from mortality. Eur J Immunol (2008) 38(12):3376–87. 10.1002/eji.200838628 19039768 PMC2749081

[24] Weiss ID Shoham H Wald O Wald H Beider K Abraham M Ccr5 deficiency regulates the proliferation and trafficking of natural killer cells under physiological conditions. Cytokine (2011) 54(3):249–57. 10.1016/j.cyto.2011.01.011 21376626

[25] Jang Y Gerbec ZJ Won T Choi B Podsiad A Moore BB Cutting edge: check your mice-a point mutation in the Ncr1 locus identified in CD45.1 congenic mice with consequences in mouse susceptibility to infection. J Immunol (2018) 200(6):1982–7. 10.4049/jimmunol.1701676 29440507 PMC5840015

[26] Legge KL Braciale TJ. Lymph node dendritic cells control CD8+ T cell responses through regulated FasL expression. Immunity (2005) 23(6):649–59. 10.1016/j.immuni.2005.11.006 16356862

[27] McGill J Legge KL. Cutting edge: contribution of lung-resident T cell proliferation to the overall magnitude of the antigen-specific CD8 T cell response in the lungs following murine influenza virus infection. J Immunol (2009) 183(7):4177–81. 10.4049/jimmunol.0901109 19767567 PMC2762786

[28] Shi C Velazquez P Hohl TM Leiner I Dustin ML Pamer EG. Monocyte trafficking to hepatic sites of bacterial infection is chemokine independent and directed by focal intercellular adhesion molecule-1 expression. J Immunol (2010) 184(11):6266–74. 10.4049/jimmunol.0904160 20435926 PMC2921650

[29] Hayakawa Y Huntington ND Nutt SL Smyth MJ. Functional subsets of mouse natural killer cells. Immunol Rev (2006) 214:47–55. 10.1111/j.1600-065X.2006.00454.x 17100875

[30] Hayakawa Y Smyth MJ. CD27 dissects mature NK cells into two subsets with distinct responsiveness and migratory capacity. J Immunol (2006) 176(3):1517–24. 10.4049/jimmunol.176.3.1517 16424180

[31] Zamora AE Aguilar EG Sungur CM Khuat LT Dunai C Lochhead GR Licensing delineates helper and effector NK cell subsets during viral infection. JCI Insight (2017) 2(10):87032. 10.1172/jci.insight.87032 28515356 PMC5436543

[32] Bernardini G Antonangeli F Bonanni V Santoni A. Dysregulation of chemokine/chemokine receptor axes and NK cell tissue localization during diseases. Front Immunol (2016) 7:402. 10.3389/fimmu.2016.00402 27766097 PMC5052267

[33] van Helden MJ de Graaf N Boog CJ Topham DJ Zaiss DM Sijts AJ. The bone marrow functions as the central site of proliferation for long-lived NK cells. J Immunol (2012) 189(5):2333–7. 10.4049/jimmunol.1200008 22821961 PMC3427014

[34] Verbist KC Rose DL Cole CJ Field MB Klonowski KD. IL-15 participates in the respiratory innate immune response to influenza virus infection. PLoS One (2012) 7(5):e37539. 10.1371/journal.pone.0037539 22624047 PMC3356330

[35] Burns DL. Subunit structure and enzymic activity of pertussis toxin. Microbiol Sci (1988) 5(9):285–7. 2908558

[36] Dawson TC Beck MA Kuziel WA Henderson F Maeda N. Contrasting effects of CCR5 and CCR2 deficiency in the pulmonary inflammatory response to influenza A virus. Am J Pathol (2000) 156(6):1951–9. 10.1016/S0002-9440(10)65068-7 10854218 PMC1850091

[37] Wareing MD Lyon AB Lu B Gerard C Sarawar SR. Chemokine expression during the development and resolution of a pulmonary leukocyte response to influenza A virus infection in mice. J Leukoc Biol (2004) 76(4):886–95. 10.1189/jlb.1203644 15240757

[38] Liu Y Zheng J Liu Y Wen L Huang L Xiang Z Uncompromised NK cell activation is essential for virus-specific CTL activity during acute influenza virus infection. Cell Mol Immunol (2017) 14:1–11. 10.1038/cmi.2017.10 28413216 PMC6203736

[39] Weizman OE Adams NM Schuster IS Krishna C Pritykin Y Lau C ILC1 confer early host protection at initial sites of viral infection. Cell (2017) 171(4):795–808e12. 10.1016/j.cell.2017.09.052 29056343 PMC5687850

[40] Jenne CN Enders A Rivera R Watson SR Bankovich AJ Pereira JP T-bet-dependent S1P5 expression in NK cells promotes egress from lymph nodes and bone marrow. J Exp Med (2009) 206(11):2469–81. 10.1084/jem.20090525 19808259 PMC2768857

[41] Martin-Fontecha A Thomsen LL Brett S Gerard C Lipp M Lanzavecchia A Induced recruitment of NK cells to lymph nodes provides IFN-gamma for T(H)1 priming. Nat Immunol (2004) 5(12):1260–5. 10.1038/ni1138 15531883

[42] Carlin LE Natural Killer Cell Activation, Trafficking, And Contribution To Immune Responses To Viral Pathogens [Dissertation Thesis]. Immunology of Infectious Diseases Common. University of Iowa (2013). Available from: http://ir.uiowa.edu/etd/1302/ (Accessed: October 7, 2014).

