# Supplementary material for: Natural Killer Cell Recruitment to the Lung During Influenza A Virus Infection Is Dependent on CXCR3, CCR5, and Virus Exposure Dose
Source: Front Immunol. 2018 Apr 17;9:781. doi: 10.3389/fimmu.2018.00781 (PMC5913326; doi:10.3389/fimmu.2018.00781)
Supplement: Supplementary file 1 [file data_sheet_1.PDF]

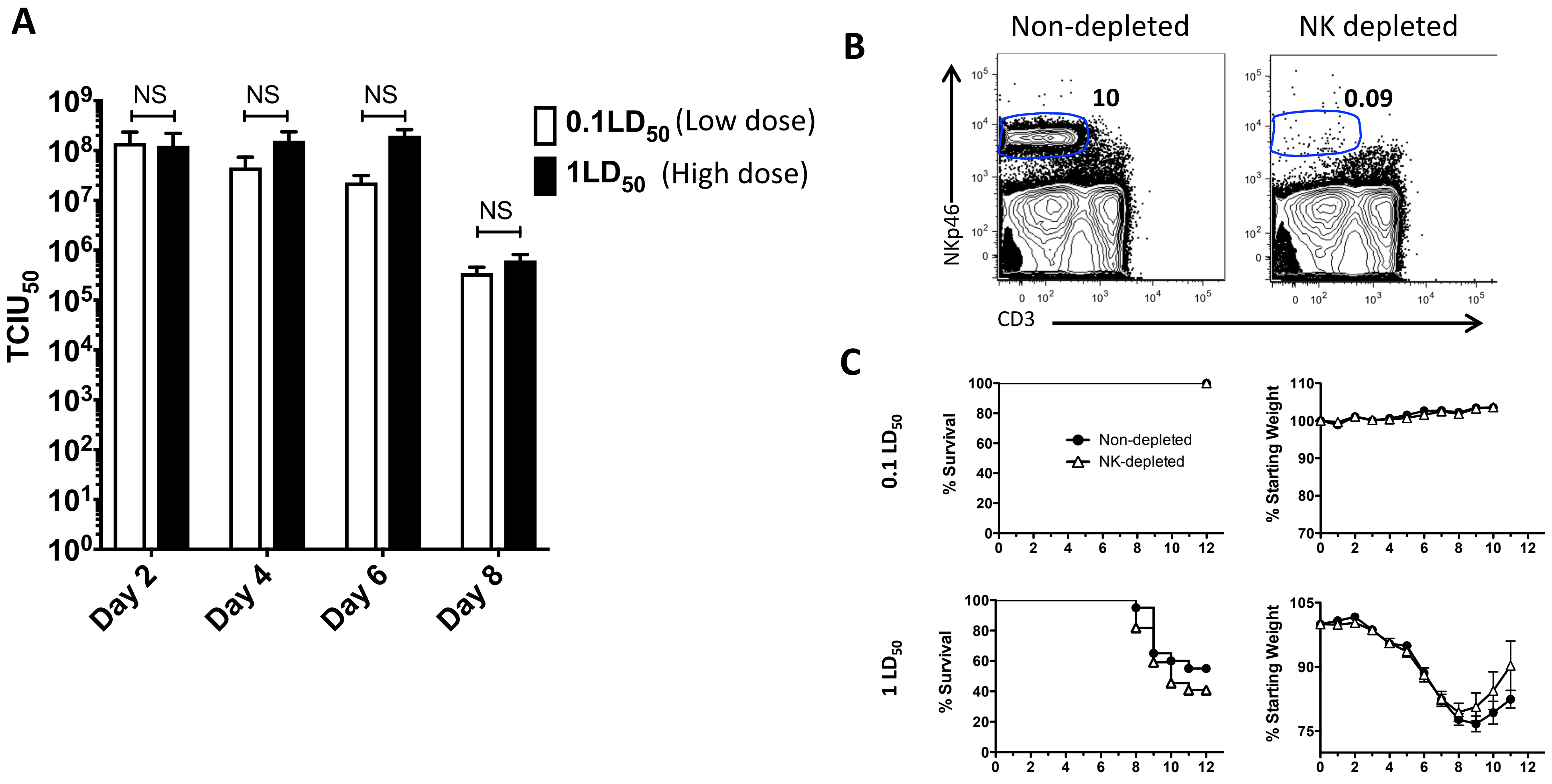

Supplemental Data Sheet 1: Depletion of NK cells does not alter IAV morbidity or mortality. (**A**) C57Bl/6 mice were infected with either a low- (0.1LD_{50}) or high- (1LD_{50}) dose of A/PR/8/34. On the indicated day post infection lungs were harvested and viral titers quantified. (**B**) CT6 mice were administered 300ug ant-NK1.1 mAb clone PK136 i.p. or left untreated (non-depleted) 7 days prior to the analysis. Lungs were harvested, and NK cells were identified as $NKp46^{+}CD3\varepsilon^{-}$ cells by flow cytometry. Data are representative of 3 experiments. (**C**) CT6 mice were treated with PK136 mAb to deplete NK cells (NK-depleted) 2 days before and on the day of infection with a low (0.1LD_{50}) or high (1LD_{50}) dose of A/PR/8/34. A group of untreated (non-depleted) CT6 mice was used as a control. Non-depleted and NK-depleted mice were monitored daily for weight loss, as a measure of morbidity, and mortality. Mice were euthanized and counted as dead upon losing more than 30% of their starting weight. Data from the 0.1LD_{50} dose represents 5 mice/group while the data from the 1LD_{50} dose was pooled from 4 independent experiments with 5 mice/group per experiment.
